# Multi–Criteria–Decision–Analysis (MCDA) for the Horizon Scanning of Health Innovations an Application to COVID 19 Emergency

**DOI:** 10.3390/ijerph17217823

**Published:** 2020-10-26

**Authors:** Matteo Ruggeri, Chiara Cadeddu, Paolo Roazzi, Donatella Mandolini, Mauro Grigioni, Marco Marchetti

**Affiliations:** 1 National Centre for HTA, Istituto Superiore di Sanità, 00161 Rome, Italy; paolo.roazzi@iss.it (P.R.); donatella.mandolini@iss.it (D.M.); marco.marchetti@iss.it (M.M.); 2 School of Medicine, St. Camillus International University of Health and Medical Sciences, 00131 Rome, Italy; 3 Department of Public Health, Universita’ Cattolica del Sacro Cuore, 00168 Rome, Italy; chiara.cadeddu@unicatt.it; 4 National Centre for Innovative Technologies, Istituto Superiore di Sanità, 00161 Rome, Italy; mauro.grigioni@iss.it

**Keywords:** horizon scanning, priority setting, Health Technology Assessment, multi-criteria decision analysis

## Abstract

*Aim:* In this article, we aim to present a tool for the early assessment of medical technologies. This evaluation system was designed and implemented by the National Centre for HTA and the National Centre for Innovative Technologies of the Istituto Superiore di Sanita, Italy, in order to respond to an institutional commitment within the “Health Technologies Assessment Team” that was established to face the huge demand for the evaluation of Health Technologies during the pandemic event caused by COVID-19, with a smart and easy-to-use framework. *Methods:* Horizon scanning was conducted through a brief assessment carried out according to the multicriteria decision analysis methodology. Each HTA domain was attributed a score according to a pros/cons and opportunities/threats system, derived from evidence in the literature. Scores were weighted according to different perspectives. Scores were presented in a Cartesian graph showing the positioning according to the potential value and the perceived risk associated with the technology. *Results:* Two case studies regarding the early assessment were reported, concerning two specific technologies: an individual protection device and a contact tracking system.

## 1. Introduction

Horizon scanning is defined as “The systematic identification of new, emerging or obsolete health technologies and potentially capable of producing effects on health, healthcare and society and which, once marketed, could have a significant clinical and economic impact on the National Health Service” [1,2,3,4].

Overall, Horizon scanning, by means of early assessments, aims to identify, with a prospective and predictive approach in the initial development phase, the health technologies that could have a significant impact on patients, public health or health systems since, in many contexts, the introduction of new technologies often takes place without prior evaluation.

Horizon scanning offers various advantages, as it guarantees the existence of a systematic approach for the identification of new or emerging health technologies. It also allows technologies to be considered for evaluation at the right time, prior to their widespread use, thus protecting patients from ineffective and potentially dangerous ones, and supporting the eligibility of a full Health Technology Assessment (HTA) process for those that, on the contrary, have shown to be innovative and potentially affordable from an economic and organizational perspective.

As a matter of fact, the decision-making processes, and in particular those aimed at acquisitions, are supported by robust assessments, conducted in compliance with the principles of transparency and the absence of conflicts of interest [1,2].

More in detail, the introduction of innovative technologies is acknowledged via a comparative assessment highlighting the additional clinical-care benefits, compared to the existing one, based on efficacy and safety data, considering needs, expected consumption volumes and costs.

In this regard, the governance of medical technologies under budget constraints aims at the efficient allocation of the resources of the National Health Service, the sustainability of innovation, the strengthening of supervision and the transparency of the actions and relationships concerning the procurement and use of medical devices. For this reason, Horizon scanning is performed by governmental agencies that provide support to single or several clients. Such systems can also be organized as networks working to achieve common goals [4].

The current health emergency caused by the COVID-19 pandemic requires high readiness on the side of researchers, decision-makers and health workers in order to quickly identify the technologies and interventions that would potentially allow, in the face of contained risks, the achievement of high added value in terms of effectivity, safety, organizational, ethical and, last but not least, economic aspects.

The last few months have seen a great multitude of technologies of all types appearing on the market (diagnostic, therapeutic, vaccines, IT platforms, applications, etc.) and at all levels, in order to find effective and efficient solutions to cope with the current crisis situation [5].

In order to support decision-makers and the funding bodies for new programs and new technologies in an informed and responsible way, it was therefore necessary to design, test and implement an evaluation system that, with a low degree of complexity and a high ease of use, was able to provide all the information for the priority setting (i.e., to identify which health technologies were potentially eligible for a further evaluation process based on the Health Technology Assessment approach).

This evaluation system was designed and implemented by the National Centre for HTA and the National Centre for Innovative Technologies of the Istituto Superiore di Sanita, Italy, in order to respond to an institutional commitment within the “Health Technologies Assessment Team” that was established to face the huge demand for the evaluation of health technologies during the pandemic event caused by COVID-19, with a smart and easy-to-use framework.

This paper aims to propose an evaluation system to carry out horizon scanning in order to support the priority setting of health technologies by identifying those potentially eligible, with particular reference to the current management of the crisis surrounding COVID 19. We also want to report two case studies focusing on the early assessment of two specific technologies: an individual protection device and a contact tracking system.

The paper includes the following parts: (1) description of the evaluation system and analysis methods used, (2) application examples, and (3) discussion.

## 2. Methods

Horizon scanning was conducted through a brief assessment carried out according to the multicriteria decision analysis methodology [6,7,8,9,10,11,12,13].

A template was used allowing a panel of experts to report the considerations regarding the characteristics of the technology on a matrix describing potential strengths/limits and threats/opportunities.

The expert panel included six members, working at the National Centre for HTA and the National Centre for Innovative Technologies of the Istituto Superiore di Sanita, Italy, with extensive experience in the field of HTA activities. Different competencies were enrolled in order to be capable of detecting the strengths/limits and threats/opportunities associated with all the different types of technologies that were expected to be considered for introduction in order to face the COVID emergency (medical devices, contact tracking apps, sanification equipment, etc.). In more detail, the panel included:

- two medical researchers with expertise in public health;
- one health economist;
- one statistician;
- one medical engineer;
- one information technology (IT) expert.

These characteristics were ordered consistently with the typical domains of the Health Technology Assessment, which included effectivity, safety, organizational, economic, legal, social and ethical aspects.

With respect to the balance between strengths/limits and threats/opportunities for each domain of the Health Technology Assessment, the expert panel assigned a score to the following: (1) the perceived value resulting from the balance between strengths and limits; and (2) the potential risk resulting from the balance between threats and opportunities. These scores ranged on a Likert scale from 1 (minimum added value or minimum risk) to 7 (which corresponded to the maximum added value or maximum risk). The total score assigned to both the value and the risk was the sum of the scores assigned to each domain of the Health Technology Assessment.

The total scores assigned were weighted according to the multi-criteria decision analysis approach, according to the analysis perspective adopted. In order to be compliant with a multi-stakeholder approach, the evaluation model allowed for considering three alternative analysis perspectives traditionally characterizing the HTA process: that of health professionals, that of decision-makers, and that of citizens/patients [7,8,9,10].

Depending on the chosen perspective, weights were used according to alternative criteria, giving more importance respectively to (1) the clinical and safety criteria in the case of health professionals, (2) the economic and organizational criteria in the case of the decision-makers’ perspectives; and (3) the ethical criteria (social desirability) in the case of the perspective of patients/citizens. The weights system (Table 1) was derived from the international literature [7,8,9,10,11,12,13,14,15]. In order to take into account the variability of the results, the weights were varied through a Monte Carlo simulation returning various scenarios. The Monte Carlo simulation was conducted assuming 1000 scenarios for each perspective [16]. The weights relating to the clinical and economic criteria (in Table 1) were associated with Beta probabilistic distributions (as per international best practices) whose related shape and scale parameters have been reported in Supplementary Table S1. The probabilistic distribution associated with the weights inherent in the social desirability criterion was instead obtained via the difference between the weights of the other two criteria, the sum being equal to one, as per construction.

Finally, the overall weights relating to the values and risks associated with the technology were arranged on a scatter plot-type graph on the ordinate axis. The risk was presented on the abscissa axis and the added value on that of the ordinates. The ratio of the risk/value of the technology could be placed in one of the following areas, as identified by the scatter plot graph:

- the area associated with a low risk and a high value (Comfort Zone) where the technologies recommended for a full HTA, with positive indication, lie;
- the area associated with a high risk and a low value in which the technologies that should be rejected are positioned (Danger Zone);
- the area associated with a high risk and a high value in which technologies are positioned whose characteristics should be studied in greater detail with a full HTA in order to formulate a final judgment (Challenge Area);
- the area associated with a low risk and a low value in which the technologies that are not a priority for decision-makers are positioned (Improvement Area).

It should be noted that the recommendation of submitting the technologies for a full HTA process was intended so as to be compliant with the methodological guidelines, and in particular with the Core Model of the European Network for Health Technology Assessment (EuNetHTA). The Core Model considers the traditional HTA domains to be addressed with a set of issues that provide a systematization of all the information collected by means of a literature review [17].

Table 2 summarizes the possible technology positioning scenarios.

## 3. Results

In the present section, two examples of early assessment referring to different technologies against COVID-19 are presented.

### 3.1. Example 1

Technology undergoing early assessment: individual protection device for COVID-19 contrast

Company: XXXX

Evaluation center: National Centre for HTA and National Centre for Innovative Technologies in Public Health, Istituto Superiore di Sanita

#### 3.1.1. Description of the Technology

The company XXXX has asked the ISS for an early assessment of an individual protection device which refers to the receipt of the application No. xxxxxxxx by the Ministry of Economic Development dated xxxxx.

This individual virus protection device consists of:

- Transparent protection device;
- Temperature and sweat sensors.

#### 3.1.2. Evaluation Results

Supplementary Table S2 shows the matrix of the perceived value/potential risk ratio that presents a description of the pros/cons and threats/opportunities of the technology being analyzed, coherent with the various dimensions of the Health Technology Assessment.

**Figure 1:**
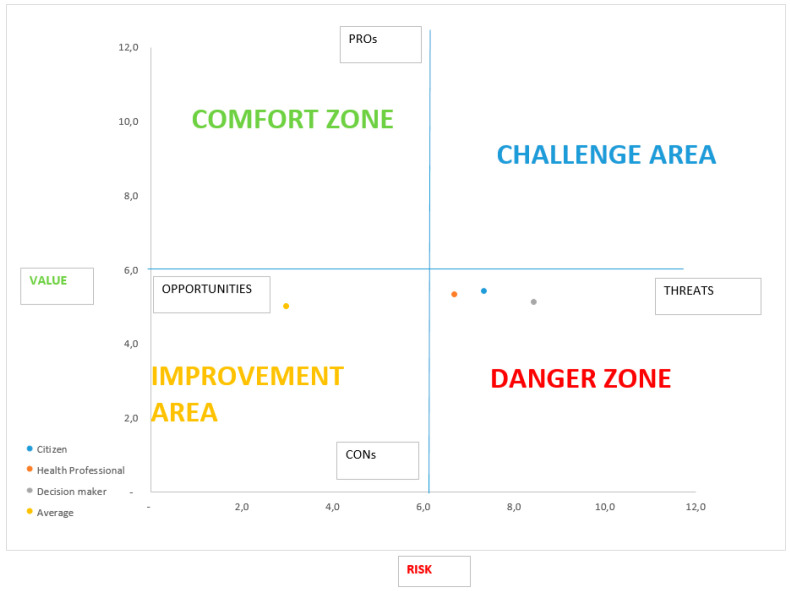
Individual protection device; perceived risk/potential value graph.

Table 3 shows the sum of the scores of each HTA domain that were attributed by the panel of experts to the perceived value and potential risk, adjusted according to the weights in Table 1. Accordingly, Figure 1 shows the positioning of the technology within the risk/value graph.

According to each perspective considered, it is possible to observe that the technology is placed in the Danger Zone, being associated with a high potential risk and a low perceived value.

Supplementary Figure S1 presents the results of the Monte Carlo simulation, which substantially confirm the results of the deterministic analysis (Figure 1) by positioning the technology in the Danger Zone in the vast majority of scenarios:

- 86% if the citizen’s perspective is considered (social desirability);
- 80% if the perspective of health workers is considered (clinical criterion);
- 90% if the decision-makers’ perspective is adopted (economic–organizational criterion).

#### 3.1.3. Recommendation

Considering all three perspectives, the technology is placed in the Danger Zone. This judgment is influenced by the high costs and organizational complexity involved in implementation. The limited advantages can be identified in the increasing safety for healthcare professionals, especially where this device replaces masks. On the other hand, however, these limited advantages are compensated for by the lower comfort and the possibility of scratching and fogging, which would increase the probability of error during maneuvers or, generally, during work.

In order to present different weighting scenarios, a probabilistic analysis with a Monte Carlo simulation was also conducted.

The results of the Monte Carlo simulation largely confirm the results by positioning, in the vast majority of the simulations carried out, the technology in the Danger Zone.

Therefore, it is recommended not to elect the technology for further assessment.

### 3.2. Example 2

Technology to be evaluated: contact tracking app

Company: XXXX

Evaluation center: National Centre for HTA and Centre for Innovative Technologies, Istituto Superiore di Sanità

#### 3.2.1. Description of the Technology

Due to the current state of emergency surrounding the COVID-19 pandemic and the necessary safety of the medical devices used in an emergency context, such as the current one, an evaluation of the contact tracking app developed by xxxxxx has been requested.

This device informs users about their probability of being exposed to COVID-19 infection, and is characterized by a complete anonymization of the data. The information is used to update the tracking capability of the device and create an area mapping that supports users in their movements by providing aggregate data.

#### 3.2.2. Evaluation Results

Supplementary Table S3 shows the matrix of the potential risk and perceived value, which presents a description of the potential, limits, threats and opportunities of the contact tracking app being analyzed, in line with the various dimensions of the Health Technology Assessment.

**Figure 2:**
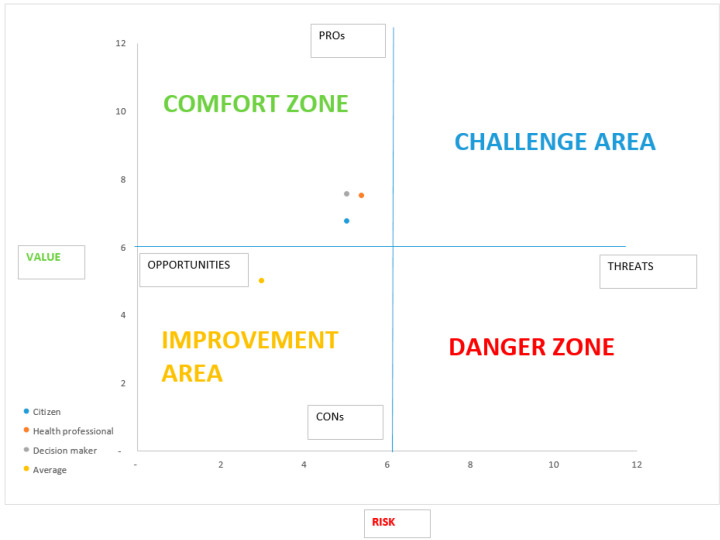
Contact tracking app; potential risk/perceived value graph.

Table 4 shows the sum of the scores of each HTA domain that were attributed by the panel of experts to the perceived value and potential risk, adjusted according to the weights in Table 1. Accordingly, Figure 2 shows the positioning of the technology within the perceived value/potential risk graph.

Considering the weighted scores for the three perspectives, it is shown that the technology is positioned in the Comfort Zone, there being associated with it a low potential risk compared to the perceived value.

Supplementary Figure S2 presents the results of the Monte Carlo simulation, which substantially confirms the results of the deterministic analysis (Figure 2.) by positioning the technology in the Comfort Zone in the vast majority of scenarios:

- 90% if the citizen’s perspective is considered (social desirability);
- 97% if the perspective of health workers is considered (clinical criterion);
- 97% if the decision-makers’ perspective is adopted (economic–organizational criterion).

#### 3.2.3. Recommendation

According to the three perspective considered, the technology is placed on the Comfort Zone, there being a high perceived value and a low potential risk associated with it. This judgment is influenced by its low costs and organizational simplicity. However, some ethical problems can be raised. It is therefore necessary to deepen the compliance of the device with the current GDPR.

For this reason, a probabilistic analysis was also conducted with a Monte Carlo simulation, so as to present different weighing scenarios.

The results of the Monte Carlo simulation largely confirm the results positioning, in the vast majority of the simulations carried out, the technology in the Comfort Zone.

For the reasons set out above, the technology is recommended to undergo full HTA and pilot testing, seeking a favorable outcome regarding compliance with the current GDPR and ease of implementation and management.

## 4. Discussion

In this paper, we presented an analytical tool to support early warning activities related to the horizon scanning of new health technologies.

The need for the designing and implementing of such tools is generated by two main factors. Firstly, there is evidence that horizon scanning is not performed with systematic and standardized methods (i.e., using checklists, stakeholders’ engagement procedures, etc., as for example in the full HTA studies).

Secondly, some disease areas, such as chronicity, oncology, etc., as well as new approaches to healthcare are experiencing a huge development and, as a result, the number of health technologies potentially eligible for reimbursement or introduction is increasing dramatically. Meanwhile, patients’ expectations are becoming increasingly complex. As a result, time to reimbursement (or introduction) calls for new approaches so as to permit rapid responses in order to avoid delays, which decrease the quality of care and health systems’ performances.

The COVID-19 experience dramatically intensified this trend, thus forcing researchers and decision-makers to increase their efforts in order to develop early warning systems, able to minimize time to decisions and manage new challenges with a matter of urgency.

Our approach has some limits. Firstly, the weights used to adjust the judgements and rankings of the expert panel were extrapolated from the literature. Of course, this paper aims to present a prototype of our horizon scanning tool, focusing on the approach more than on the results of single assessments. However, future research will be focused on eliciting context-specific preferences, using discrete choice experiments or other contingent evaluation frameworks in an Italian sample including health professionals, patients and decision-makers.

Secondly, our ranking scales were based on the Likert methodology, which is the most-used approach when switching from qualitative judgements to quantitative assessments. However, our choices for setting up the frontiers of the quadrant (see Figure 1 and Figure 2) were arbitrary (the origin of our axes were set to 3.5, the average of the Likert scale ranging 1–7). Further research is needed in order to provide a more robust rationale to set up and calibrate the quadrant.

Thirdly, the expert panel included professionals within the COVID Team, thus inducing a selection bias. A systematic approach is needed in order to select experts. This is particularly important since, when the flux of new health technologies is massive and extremely differentiated (as in the case of the COVID-19 emergency), a higher number of experts is needed either to avoid excessive workloads or to improve the ensemble of available competencies for assessing different types of technologies.

## 5. Conclusions

This paper showed how an early assessment of health technologies can be a useful tool to support the priority setting process when the decision making is under pressure due to unexpected events of situations of crisis. The COVID - 19 pandemics is an interesting field of application either for the number and different types of health technologies potentially eligible for introduction, and the amount of resources involved in the management of the emergency.

## Figures and Tables

**Table 1:** Weights assigned to the different perspectives (Standard deviation).

Perspective	Criteria		
	Economic (SD)	Clinic (SD)	Ethical (SD)
Patients/Citizens	15% (5%)	45% (12%)	40% (9%)
Health Professionals	30% (11%)	40% (10%)	30% (6%)
Decision-makers	35% (7%)	35% (8%)	30% (6%)

**Table 2:** Technology positioning scenario.

Positioning	Risk	Value	Scenario	Recommendation
Comfort Zone	Low	High	No negative scenario identified	Full HTA with positive indication
Improvement Area	Low	Low	Limited value, not a priority	Reject with chance of further evaluation when additional information is provided
Danger Zone	High	Low	Low potential value for money	Reject
Challenge Area	High	High	Promising technology, deserves further research in order to estimate the value for money	Full HTA report

**Table 3:** Individual protection device: scores for each perspective and criteria.

**Potential Value**				
**Perspective**	**Criteria**			**Total**
	**Economic**	**Clinical**	**Ethical**	
Patients/Citizens	0.60	3.60	1.20	5.40
Health Professionals	1.20	3.20	0.90	5.30
Decision-makers	1.40	2.80	0.90	5.10
**Perceived Risk**				
**Perspective**	**CRITERIA**			**Total**
	**Economic**	**Clinical**	**Ethical**	
Patients/Citizens	1.80	3.15	2.40	7.35
Health Professionals	2.10	2.80	1.80	6.70
Decision - makers	4.20	2.45	1.80	8.45

**Table 4:** Contact tracking app, scores for each perspective and criteria.

**Potential Value**				
**Perspective**	**Criteria**			**Total**
	**Economic**	**Clinical**	**Ethical**	
Patients/Citizens	1.50	4.05	1.20	6.75
Health Professionals	3.00	3.60	0.90	7.50
Decision-makers	3.50	3.15	0.90	7.55
**Perceived Risk**				
**Perspective**	**CRITERIA**			**Total**
	**Economic**	**Clinical**	**Ethical**	
Patients/Citizens	0.75	2.70	1.60	5.05
Health Professionals	1.80	2.40	1.20	5.40
Decision-makers	1.75	2.10	1.20	5.05

## Supplementary Materials

The following are available online at https://www.mdpi.com/1660-4601/17/21/7823/s1, Figure S1: Individual virus protection device, Monte Carlo results, Figure S2: Contact tracking device, Monte Carlo results, Table S1: Scale and Shape parameters used in Monte Carlo Simulation, Table S2: Individual virus protection device, Potential Risk- Perceived Value matrix Table S3: Contact tracking device, Potential Risk—Perceived Value matrix.
