# Supplementary material for: Multi–Criteria–Decision–Analysis (MCDA) for the Horizon Scanning of Health Innovations an Application to COVID 19 Emergency
Source: Int J Environ Res Public Health. 2020 Oct 26;17(21):7823. doi: 10.3390/ijerph17217823 (PMC7662805; doi:10.3390/ijerph17217823)
Supplement: Supplementary file 1 [file ijerph-17-07823-s001.pdf]

SUPPLEMENTARY MATERIALS

Supplementary Table S1. Scale and Shape parameters used in Monte Carlo Simulation

Parameters	Economic criteria	Clinical criteria
Scale	0,23	0,47
	0,27	0,56
	0,78	0,64
Shape	1,31	0,58
	0,63	0,84
	1,46	1,19

Supplementary Figure S1. Individual virus protection device

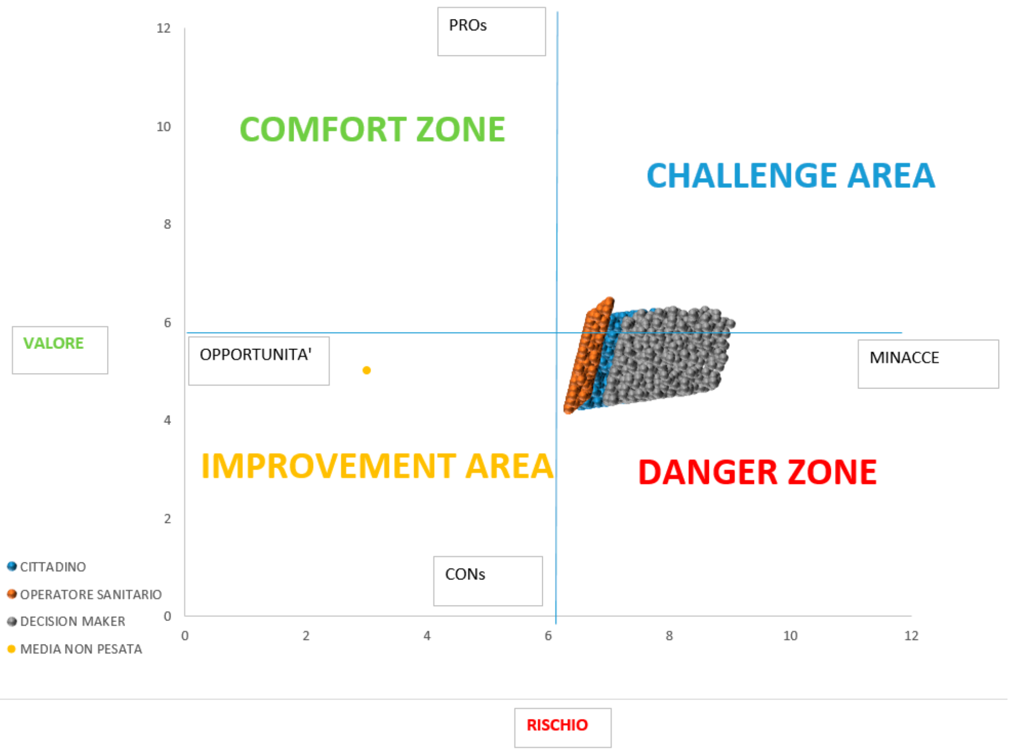

Supplementary Figure S2. Contact tracking device, Monte Carlo results

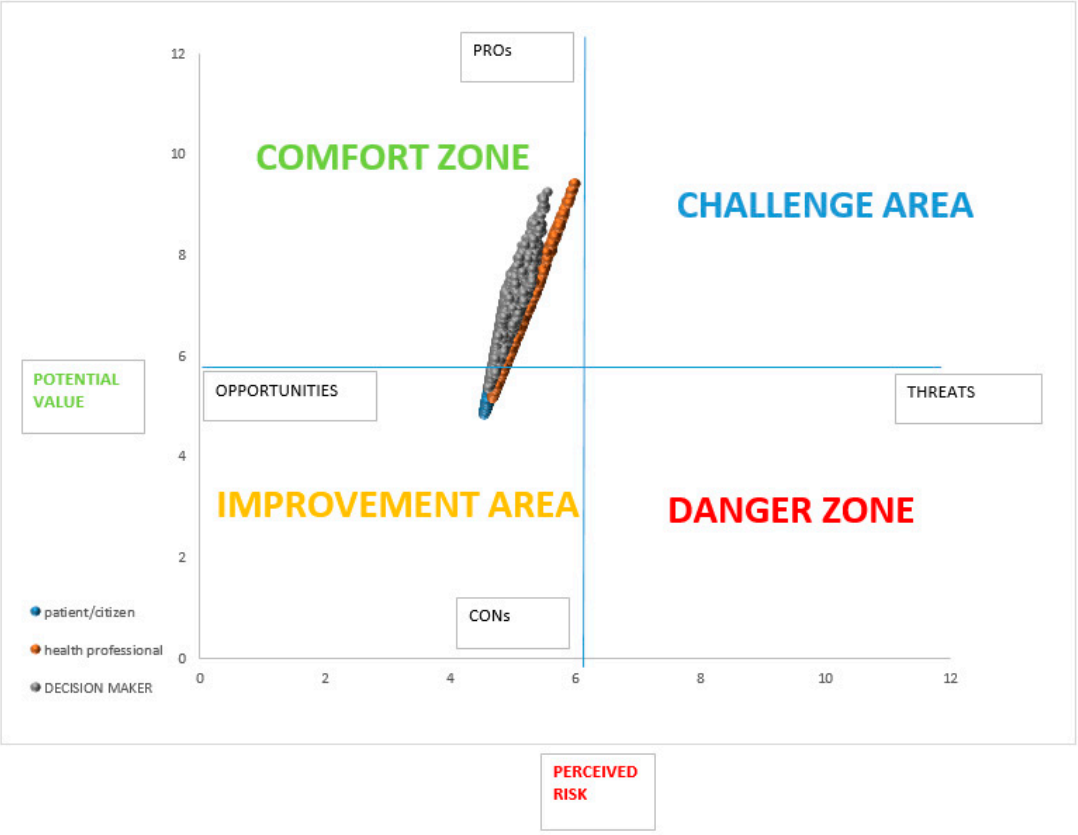

Supplementary Table S2. Individual virus protection apparatus, Potential Risk- Perceived Value matrix

Domains	Pros	Cons	Threats	Opportunities
**Effectiveness**	• continuous monitoring of temperature<br>• integrated protection: mouth, nose and eyes	• no back up in case of malfunctionings<br>• uncomfortable	• stress of health professionals	• chance of immediate actions in case of alert<br>• no need for masks
**Safety**	• no need for filters (can cause damages and toxicities if substitution is delayed)<br>• no signs and bruises of health professionals faces	• problems in wearing the devices in case of fire or other emergencies<br>• chance of fogging and scratching	• stress<br>• risk for health professionals health in case of evacuation (ie fire)<br>• chance of clinical errors iin case of scratching or fogging	• social distancing is more fficient
**Organizational aspects**	• no need for breaks in order to measure and monitor temperaure	• immediate action in case of alert challenging<br>• need for space to recharge batteries and store helmets<br>• need for personnel for sanification and substitution damaged helmets	• risk of unjustified alters can decrease productivity and slow time to respnse during health emergencies<br>• stress and burn out due to continuous work and continuous monitoring	• no breaks to measure temperature can increase efficiency

**Economic aspects**	• multi-use device<br>• multi-year mortage	• not sure if useful after covid emergency<br>• high cost due to energy sanification, storage…<br>• high opportunity costs if compared with other alternatives (i.e. temperature monitoring every 2 hours)	• waste of resources<br>• not cost effective<br>• increase of inefficiency of health expenditure	• costs can be partially compensated with the decrease of costs and space for storage of masks
**Ethics, Social and legal aspects**	• positive social externality	• stigma<br>• disciplinary measures difficult to identify and to apply in case of non adherence	• increase of stress due to stigma	• positive signal and social commitment

Supplementary Table S3. Contact tracking device, Potential Risk – Perceived Value matrix

Domains	Pros	Cons	Opportunities	Threats
**Effectiveness**	• standard platforms needed	• effectiveness depends on the number of people using the technology		• low adherence<br>• chance of biases in upgrading registers
	• risk of contact is traced, not people	• volounteering base<br>• only IoS techology is allowed	• if massive use is achieved, lockdowns can be shorter	
	• doesn’t trace mobility on time	• available informative matherial is scarce<br>• detecting range higher than bluetooth is not a necessity in order to reduce COVID contagion		
	• it uses the mobility history in order to predict contact<br>• data already collected for other uses<br>• doubles the chance od detecting infected comparedd to bluetooth			
**Safety**	• anonymized data<br>• cripted data	• private data management	• users feel not to be surveilled<br>• minimize risk of biased information	• excessive trust can bring to under-evaluation of non-mapped risks<br>• risk of hackerage

		• not clear compliance with GDPR		
**Organizational aspects**	• data storage with already existing technologies<br>• no need to download app<br>• fast upgrade	• need to manage hackerages or malfunctioning<br>• volounteer base<br>• poor available informative matherial on functioning and safety.	• Fast development and implementation<br>• technology available for massive use	• patients not adherent<br>• malfunctioning can cause biases in thee contact tracing and increase of contagiousness
**Econmics**	• affordable costs of implementation<br>• low manteinance costs	• need incentive to increase adherence<br>• not clear if use is free of charge	• decrease of lockdwn duration<br>• increase of social welfare	• if not free of charge chance of poor effectiveness and increase of inequalities
**Ethics, Social and Legal aspects**	• no surveillance on time<br>• no individual tracing	• surveillance not completely avoided<br>• mapping of entire areas , stigma<br>• poor informative matherial available	• incentive to social cooperation and commitment<br>• increase on capabilities<br>• chance of taking more informed and responsible choices	• social stigma (entire areas or categories)<br>• inequalities increase if not free of charge
